# Periprosthetic Joint Infections of the Knee—Comparison of Different Treatment Algorithms

**DOI:** 10.3390/jcm13133718

**Published:** 2024-06-26

**Authors:** Hans-Robert Springorum, Clemens Baier, Günther Maderbacher, Alexander Paulus, Joachim Grifka, Juergen Goetz

**Affiliations:** 1 Orthopädisches Fachzentrum Weilheim-Garmisch-Starnberg-Penzberg, University Hospital Regensburg, 93077 Bad Abbach, Germany; 2 University Hospital Regensburg, 93077 Bad Abbach, Germany; 3 Ludwig-Maximilians-Universität München, 81377 München, Germany; 4 Orthopaedic University Hospital Regensburg, 93077 Bad Abbach, Germany; 5 Medartes Praxisverbund Regensburg Neutraubling, University Hospital Regensburg, 93077 Bad Abbach, Germany

**Keywords:** periprosthetic joint infection (PJI), total knee arthroplasty, joint aspiration, re-infection rate, revision surgery

## Abstract

**Background:** Periprosthetic joint infection (PJI) following total knee arthroplasty is a serious complication lacking evidence-based diagnostic and treatment protocols, particularly in ruling out persisting infection before reimplantation. **Methods:** This retrospective analysis assessed the mid-term outcomes of 66 patients undergoing septic two-stage knee revision surgeries from 2007 to 2013, diagnosed as per the Musculoskeletal Infection Society criteria. After implant removal and antibiotic treatment, reimplantation decisions were based on either joint aspiration, blood counts, and clinical examination (group A) or an open biopsy (group B). Both groups underwent meticulous debridement and spacer exchange during the interim period. **Results:** Late re-infection occurred in 12.1% of all patients. In group A, 13.8% experienced late re-infection, with 14.3% in subgroup A1 and 13.3% in subgroup A2. In group B, 10% had a late re-infection. No significant difference in re-infection or complication rates was found between the groups. **Conclusions:** The study did not demonstrate the superiority of group B’s approach of open biopsy over group A’s joint aspiration, clinical examination, and blood counts in preventing re-infection or reducing complications.

## 1. Introduction

Increasing numbers of primary total knee arthroplasty in industrial countries will cause growing numbers of revision total knee arthroplasty within the next few years [1]. The reasons for revision surgery are various [2]. One of the most challenging problems after total knee arthroplasty is periprosthetic joint infection (PJI). The incidence ranges from 0.4% to 4.0% [3,4].

Periprosthetic joint infections are associated with high morbidity due to the long duration of treatment and severe mental and physical stresses [5]. It can result in loss of function or even limb amputation, with a permanent loss of quality of life [6]. Furthermore, cases of PJI are associated with high socioeconomic costs [7].

The mode of infection is either exogenous or hematogenous. Exogenous infections typically occur in the early postoperative period, and patients with multiple comorbidities are prone to developing such postoperative complications [8]. Periprosthetic joint infections are commonly classified into four types (see Table 1) [9].

McPherson et al. described a further classification, including three categories: infection type (acute versus chronic), systemic host grade, and local extremity grade.

In the guidelines of the Infection Disease Society of America (IDSA) [10], the American Academy of Orthopedic Surgeons (AAOS) [11], the International Consensus on PJI [12,13] and the Liestal Algorithm from Switzerland of Mauerer and Ochsner [14], there is a consensus for the differentiation between early and late infection at 3 to 12 weeks after surgery or the development of symptoms in cases of hematogenous late infection [15].

The risk factors for periprosthetic joint infections are male sex and low social status [3], as well as diabetes mellitus (HbA1c > 7), malnutrition, obesity (BMI > 40 kg/m^2^), chronic renal failure, surgical procedures in the short past, active liver disease, smoking, alcoholism, intravenous drug abuse, posttraumatic arthritis, inflammatory diseases of the joints, long-term cortisone therapy, hypoalbuminemia, previous surgical interventions of the affected joint, and severe immune disorders [16]. In some studies, chronic lung diseases, coagulopathy, heart diseases, depression, and metastatic cancers with a higher ASA-Score, the transfusion of foreign units of blood, and urinary tract infections are mentioned as risk factors as well [3,17,18,19].

A two-stage joint replacement algorithm is applied in most patients diagnosed with late PJI. However, the management of PJI is still not standardized, nor is there an international evidence-based consensus, although there are useful recommendations for diagnostic measures and treatment [11,15,20,21,22]. Most knowledge about the outcome of PJI, is derived from observational studies. For a favorable outcome of PJI, surgical management as well as antimicrobial therapy are both crucial factors [8].

In late infections, the standard of treatment is implant removal and radical surgical debridement in combination with antibiotic therapy [10]. Before re-implantation, persistent infections should be ruled out. There are different options to examine the healing process. Blood counts (CRP, BSR, IL-6, WBC), joint aspiration, as well as microbiological cultures and histological analysis of periprosthetic tissue, are the most common ones [20].

The main problem for PJI surgeons is knowing when the right time for reimplantation is and when the infection has been adequately treated so that the replacement can be successful in terms of re-infection rate.

In this study, two different strategies of treatment for late PJI of the knee were examined retrospectively. We hypothesized that an open biopsy for the extraction of histological samples and cultures provides a better patient outcome compared to a mere joint aspiration before re-implantation surgery regarding the target criteria of re-infection. Open sample collection is compared to aspiration and blood values.

## 2. Materials and Methods

This study is a retrospective analysis of 66 patients with septic two stage revision surgeries of the knee in our department from 2007 to 2013. All patients treated with the diagnosis of late periprosthetic joint infection of the knee were registered. Exclusion criteria were treatment with arthrodesis and patient follow-up of less than 12 months. Patient records were used for data analysis as well as a telephone interview with the patients and their family doctor.

### Treatment Algorithm

Periprosthetic joint infections were diagnosed according to the definitions of the Musculoskeletal Infection Society (MSIS) that were used in our department already before publication in 2013. Late and chronic periprosthetic joint infections were treated with a two-stage procedure with implant removal, meticulous debridement and synovectomy, instillation of polyhexanid, a PMMA spacer containing antibiotics, drainage, and systemic antibiotic administration for 6 weeks according to the resistogram or calculated with either moxifloxacin and rifampicin (first line treatment), amoxicillin/clavulanic acid and rifampicin (second line), or vancomycin and rifampicin (third line). The PMMA spacer contained gentamycin and, in proven and sensitive MRSA/MRSE infections, also vancomycin [23].

Six cultures were taken intraoperatively and kept for 14 days, along with at least 2 biopsies for histological analysis, according to the findings of Schaefer et al. [24].

After 6 weeks, antibiotic administration was stopped for 2 weeks, and reexamination was performed for verification of a cured infection:

Group A:

Till 2009, joint aspiration was performed in an operating room, and WBC counts of the synovial fluid and cultures were taken and kept for 14 days.

(Subgroup A1, *n* = 21)

If no cultures were obtained (dry aspiration or renunciation by the surgeon), a decision for re-implantation was made according to clinical examination and blood cell counts.

(Subgroup A2: *n* = 15)

Group B:

From 2009, the treatment algorithm was changed in order to improve the diagnostic sensitivity of persisting joint infections. Therefore, an open biopsy was performed. The surgical treatment was comparable to the implant removal procedure with 6 microbiological cultures, 2 biopsies, meticulous debridement, the instillation of polyhexanid, the exchange of spacers, drainage, and wound closure. Systemic antibiotic therapy was given after sample collection according to the resistogram of the infecting organism detected during implant removal. If the cultures were positive, antibiotic therapy was planned again for 6 weeks according to the resistogram. If the cultures were negative, re-implantation was planned as a subsequent operation as soon as possible, depending on the soft tissue conditions. (Group B: *n* = 30)

Statistics:

Statistical analysis was performed with Microsoft Excel (version 2019 MSO, Microsoft Corporation, Seattle, WA, USA).

## 3. Results

### 3.1. Demographic Data

The average age at diagnosis of all patients was 64.6 years (SD ± 9.8; min = 34, max = 87), in group A 62.9 years (SD ± 9.6), and in group B 66.5 years (SD ± 9.6). The female patients had an average age of 66.4 years, and the male patients’ average age was 63.4 years. There were 41 (62.1%) male patients and 25 (37.9%) female patients in both groups; in group A, there were 23 (63.9%) male patients, and in group B, there were 18 (60.0%) male patients.

### 3.2. Follow-Up Period

The average follow-up period of all 66 patients was 49.4 (SD ± 23.0; min = 12, max = 92) months. In group A, the follow-up period was 61.3 (SD ± 20.3) months; in group B, the follow-up period was 35.9 (SD ± 17.6) months. The difference in both groups is related to the change of diagnostic procedure before re-implantation in 2009 in our department. Therefore, the follow-up period is longer in group A.

### 3.3. Interval/Time Period from Total Knee Arthroplasty to Diagnosis of Infection

The interval from total knee arthroplasty (respectively, the last operation at the affected knee with negative cultures and negative histological findings) to the diagnosis of late infection was 29.1 (SD ± 43.8) months. In group A, the average was 27.8 (SD ± 39.8) months, and in group B, it was 31.4 (SD ± 49.0) months.

### 3.4. Interval/Time Period from Implant Removal to Re-Implantation

The interval from implant removal to re-implantation in the course of the two-stage procedure was median 121 (min 77; max 333) days. In group A, the median interval was 121 (min: 77; max 177)) days, and in group B, it was 138 (min: 77; max:333) days (*p* = 0.16).

### 3.5. The Infecting Organisms at Diagnosis Respectively Implant Removal

The frequency and distribution of infecting organisms in both groups and the total number are shown in Table 2.

### 3.6. The Infecting Organisms at Joint Aspiration Respectively at Open Biopsy

Group A:

The infecting organisms in group A are focused on group A1 (21 patients) with a successful joint aspiration before re-implantation. No positive cultures were found in all aspirates (0%) after 14 days of incubation. Three patients had a second joint aspiration due to clinical symptoms of persisting infection, again without positive cultures. Nine patients in group A1 had an additional open biopsy with spacer exchange (two for worsening of blood counts and clinical examination, two for wound healing problems, one for intraoperative appearance of persistent infection, and four for not further specified reasons), but all cultures were negative in these open biopsies.

Group B:

Out of 30 patients, 18 showed a negative culture at the open biopsy.

Positive cultures were found in 12 (40.0%) patients, in four patients, the initial isolated organism of implant removal was found, in one patient with a mixed infection only one of the previous infecting organisms was found; and in seven patients, another organism was monitored. Details are presented in Table 3.

Due to the positive cultures in these 12 patients, a second therapy interval with 6 weeks of systemic antibiotic administration and a second sample was collected after discontinuing antibiotic therapy for 2 weeks. In three more patients, a second sample collection was performed because of elevated blood counts and a clinical examination.

Seven (46.7%) of these patients showed negative cultures, eight (53.3%) of these patients had positive cultures again, and two of them showed negative cultures in the first sample collection but positive blood counts and clinical examination. In these eight patients, there were three changes of organism. Details can be found in Table 4.

These eight patients and two patients with negative cultures (due to clinical examination and blood counts) received a third treatment interval with systemic antibiotic administration according to the resistogram as mentioned above, and after that, they discontinued antibiotic therapy for 2 weeks. In six of these patients, cultures were negative at the third sample collection, and they received a total knee arthroplasty. One of these eight patients had a joint aspiration with a negative culture and was reimplanted. One patient insisted on reimplantation despite positive cultures. Two patients had one, and two, respectively, more therapeutic cycles with sample collection before reimplantation.

Fifteen (50%) patients in group B had a re-implantation after the first sample collection.

### 3.7. The Infecting Organisms at Re-Implantation

In 16 (24.2%) of the 66 patients altogether, positive cultures were monitored at re-implantation.

Group A showed a positive culture in eight (22.2%) patients, 6 (28.6%) patients in group A1 and two (13.3%) patients in group A2); thus, 6 of 21 (28.6%) patients following joint aspiration showed false negative results.

Group B showed a positive culture in eight (26.7%) patients. Details of all positive cultures can be found in Table 5.

### 3.8. Re-Infection Rate and Infecting Organisms

The overall late re-infection rate in all 66 patients was 8 (12.1%) in the follow-up period.

In group A, there were five (13.8%) patients with a late re-infection, 3 of 21 (14.3%) in group A1 and 2 of 15 (13.3%) in group A2. In group B, there were three (10%) patients with late re-infections in the follow-up period. Details can be seen in Table 6.

If the follow-up period of both groups is adjusted (reduced follow-up in group A) to the mean follow-up period of group B (35.9 months), there were only four patients with a late re-infection in group A (11.1%). No significant difference could be seen in the re-infection rate between the two groups (*p* = 0.43).

There was no significant difference in accuracy for predicting the probability of reinfection between the two groups.

The median duration of late re-infection after re-implantation was 7 months (min:1; max:58); in group A, the median was 8 (min: 5; max:58) months, and in group B, the median was 3 months (min:3; max: 21).

Group A

At the time of re-infection in group A1, different infecting organisms were monitored in two patients; in one patient, the formerly seen organism was found. In group A2, both cultured organisms were the same that were already detected at the time of implant removal. In one of these patients, the infection could not be cleared, and after another attempt at limb preservation with a two-stage revision surgery, the patient ended up with an above-knee amputation.

Group B

At the time of re-infection in group B, different organisms compared to the initial infection, sample collection, and re-implantation were monitored in two patients. In one patient, infection could not be controlled, and arthrodesis as a salvage procedure was performed.

### 3.9. Complications

Beside the late re-infections, 21 (31.8%) patients suffered complications. In group A, there were 12 (33.3%) patients, and in group B, there were 9 (30%) patients. There was no significant difference between both groups (*p* = 0.93).

Ten patients suffered from wound healing problems with superficial wound revision (six patients), vacuum assisted closure therapy (three patients), or a gastrocnemius flap with a skin mesh graft (1 patient).

Group A:

Four patients had relevant wound healing problems; two patients suffered from arthrofibrosis; one patient had an arterial occlusion and was successfully treated with lysis therapy; one patient suffered from deep vein thrombosis; one had a transient ischemic attack (TIA) after implant removal; one suffered from dislocation of the knee and received a hinged implant; one patient fell and had a rupture of his patella ligament, which was surgically treated; and one patient had temporarily cardiovascular problems and an erysipelas at the affected lower limb.

Group B:

Six patients suffered from relevant wound healing problems; one patient had an arterial occlusion 4 weeks after reimplantation and received an endovascular stent; one patient had a decubitus at the heel; one patient suffered from acute hemolytic-uremic syndrome after antibiotic treatment with vancomycin; one patient suffered from dislocation of the PMMA-spacer, which lead to revision surgery; one patient left the department with the suspected diagnosis of reinfection against medical advice; and one had severe sepsis that ended in above-knee amputation.

Details can be seen in Table 7.

## 4. Discussion

The objective of this study was a comparison of different treatment algorithms for late periprosthetic joint infection of the knee. A persistent infection of the affected joint should be excluded before re-implantation. In group A, the exclusion criteria for persistent infection were a clinical examination, blood counts (subgroup A2), and in subgroup A1, an additional joint aspiration. In group B, an open biopsy with the collection of six microbiological cultures and at least two histological samples in combination with an exchange of the PMMA spacer was undertaken. The main finding was that the re-infection rate was 12.1% in all patients and did not show significant differences between both groups (group A with 13.8% and group B with 10.0%).

Although patients in group B were longer hospitalized and were operated on at least once more, the outcome regarding re-infection rate or complications was not better. Taking into account the shorter follow-up period in group B, the results regarding these parameters were almost the same. If the follow-up period of group A was adapted to group B, the re-infection rate was more or less the same, with 10% in group B and 11.1% in group A.

For the periprosthetic joint infection, there is still no golden standard of treatment established. There is a consensus for the differentiation of early and late infections and for implant removal in late infections. Many other treatment options are still not standardized.

Should the exchange arthroplasty be undertaken in a one-stage or two-stage procedure? Should a mobile or fixed PMMA spacer be used, and should it be exchanged after a defined period of time? What kind of antibiotics should be administered locally and systemically, and for how long? What is the most effective preoperative method to exclude persistent infections? Is it clinical examination and blood counts only, or also a joint aspiration or even an open biopsy with spacer exchange for cultures and histological analysis?

According to the literature a two-stage surgical strategy is widely preferred [25,26,27,28,29]. However, the incidence of re-infection (persistent or recurrent) after a one-stage exchange arthroplasty might not be higher. The incidence of re-infection is reported at 9–33% in one-stage as well as in two-stage procedures [30,31,32,33,34,35,36,37,38,39].

The reinfection rate in this study was in the same range, with 8 out of 66 patients (12.1%) in the follow-up period altogether. Comparative studies with a high level of evidence are not available [15].

Before one-stage or two-stage exchange arthroplasty, the infecting organism should be identified either by joint aspiration or by an open or arthroscopic biopsy. The identification of the infecting organism and its resistogram allows an effective and special antibiotic treatment, peri- and postoperatively. Joint aspiration is the most commonly used test for identification. However, the literature shows sensitivity from 12–100% and specificity from 81–100% [40,41]. This is comparable to our study population, which had negative results after all joint aspirations and positive microbiological samples at re-implantation in six patients.

Klaber et al. reported that the sensitivity and specificity of open biopsy were 69.4% and 89.1%, respectively [42].

Besides the removal of the implant, including the bone cement, meticulous and extensive debridement and irrigation must be carried out in proven late periprosthetic infections. Irrigation according to the protocol of the International Consensus Group is recommended [43]. The bone cement used for the antibiotic-loaded spacer and, respectively, for the exchange arthroplasty should refer to the resistogram of the identified organism [10,14,44,45].

Comparing both one-stage and two-stage revision arthroplasty, there are advantages and disadvantages:

A one-stage treatment algorithm implies only one surgical procedure, reduced hospitalization time, reduced overall costs, shorter systemic antibiotic administration, and relatively improved patient satisfaction [10,31,46,47,48,49].

The contraindications for a one-stage exchange arthroplasty are mentioned by Gehrke et al. [31]: sepsis, failure of two previous one-stage procedures, infection involving the neurovascular bundles, an unknown infecting organism and its resistogram, infection with a highly virulent organism, and extensive soft tissue involvement preventing closure of the wound [15].

A two-stage treatment algorithm allows for increased time of antibiotic impaction, offering the affected joint enough time to heal with a high local concentration well below systemically toxic levels [50,51]. Remaining organisms after irrigation and debridement can be killed due to the high elution of antibiotics [15,52,53]. The PMMA spacer also maintains limb length, prevents contractures, and might improve exposure during the second-stage procedure. In the second stage, there is the opportunity for a second meticulous debridement, knowing well the most affected areas of the infected joint from microbiological and histological analysis.

In this study, articulating self-made spacers were used in order to obtain at least a minimum of function, muscle strength, and mobility for the patients. There is no evidence that self-made or manufactured spacers, articulating or non-articulating spacers, achieve better results [13,54]. One should also bear in mind that PMMA is an abrasive substance and may interfere with the surrounding soft tissue.

Prior to re-implantation, persistent infection should be ruled out with at least a clinical examination and blood counts (ESR, WBC, CRP) although patients with seronegative PJIs are known in up to 4% of the population [55]. In the joint aspiration group, none of the joint aspirations taken prior to second-stage exchange arthroplasty showed positive cultures, although at re-implantation, six patients showed positive cultures. However, the joint aspiration was performed with the enclosed antibiotic-eluting PMMA spacers. According to the literature, the eluting period is up to 6 months, with a fast decrease in released antibiotics within several days [50,56]. Therefore, the detection of infecting organisms might have been limited. The sensitivity of joint aspiration in the presented study (group A1) was poor, at 0%, although cultures were kept for 14 days. In 6 of 21 patients (28.6%), joint aspiration showed a false negative result. Boelch et al. found a sensitivity and specificity of synovial fluid culture of 4.6 and 94.3% in patients with joint aspiration during the two-stage exchange of the hip with spacers [57].

Fink et al. recommended biopsies to improve sensitivity and specificity. In this study (group B), 8 patients out of 15 (53.3%) were false-negative with negative biopsies at sample collection and positive cultures at re-implantation. Fink et al. did not observe false negative results, but they had two patients with false positive results with biopsies [40].

The isolated organism of 7 out of 12 patients (58.3%) in group B with a positive culture at first sample collection was different from the previously identified organism at implant removal. At the second sample collection three out of eight patients (37.5%) showed a different organism than in the first sample collection. Azzam et al. reported only 1 of 17 patients (5.9%) in their population. We partly attribute these results to our perioperative antibiotic treatment at open biopsy. The surgical procedures were started without single-shot antibiotic therapy. Antibiotic treatment was started as recently as the sample collection was finished. To eliminate this gap, since 2014, our patients have received standard perioperative antibiotic treatment with a single-shot dosage approximately 30 min up to 1 h prior to surgery, according to the protocol of Tetreault [58]. They found no effect on the results of cultures obtained intraoperatively when prophylactic antibiotics were administered before surgery.

The optimal time interval for second-stage re-implantation is not clear yet. In most treatment algorithms, a re-implantation is performed 6 to 12 weeks after implant removal [14,15,53,59]. We performed re-implantation in the joint aspiration group at an average interval of 17.7 weeks (SD ± 6) and in the open biopsy group after 22 weeks (SD ± 10). The reasons for delay were poor medical condition, missing compliance of the patient, unsatisfactory clinical examination or increasing blood counts, awaiting the final histopathological and microbiological results, a second joint aspiration, the intraoperative suspicion of persistent infection, wound healing problems, or several open biopsies.

## 5. Limitations

This study also has certain limitations. First of all, these are the retrospective study design and the number of patients. The authors considered increasing the number of cases and including more recent cases in the analysis. However, since the treatment regime was not changed after 2009, extending the evaluation period would only have increased the number of cases in one group and would therefore not have improved comparability between the two groups. Regarding the literature, high sample size studies dealing with PJI are rare and usually have a retrospective design.

The patient population in our study is heterogeneous, the infecting organisms are various. Therefore, the antibiotic treatment was different between the patients.

The follow-up period for both groups was not consistent due to the change in treatment algorithm in our department in 2009.

## 6. Conclusions

This study did not show an advantage for group B with open biopsy and spacer exchange prior to re-implantation regarding re-infection and complication rate compared to group A with clinical examination and blood counts (group A2), respectively, complemented with joint aspiration (subgroup A1). The higher effort in group B with an open biopsy was associated with increased morbidity, higher costs of treatment, a longer duration of treatment, and immobilization.

Joint aspiration with an enclosed spacer was not helpful for the detection of persistent infections.

The surgeon’s experience with the preoperative and intraoperative assessments is still an important factor in the determination of therapy. The detection of a reliable biomarker confirming or excluding PJI is a desirable goal for the diagnostic and therapeutic progress of PJI. An additional tool to improve these results might be sonification, which should be implemented in the diagnostic process.

## Figures and Tables

**Table 1:** Classification of periprostethic joint infections by Tsukayama [9].

Type	Infection
I	positive culture at exchange arthroplasty
II	early postoperative infection (<4–6 weeks)
III	acute hematogenous infection
IV	late infection (>6 weeks)

**Table 2:** Distribution of the identified organisms at implant removal in both groups and in total.

Infecting Organisms	Group A	Group B	Total
Staphylococcus *spp.* Coagulase negative (CNS) *	14	10 *	24
Staphylococcus aureus **	4	4 **	8
*Staphylococcus epidermidis*	3	3	6
*Enterococci*	3	1	4
*ß-haemolytic Streptococci group B*	3	1	4
*Streptococcus viridans (not further classified)*	-	2	2
*Staphylococcus capitis*	-	2	2
*Propionibacterium acnes*	1	1	2
*Staphylococcus lugdunensis*	1	1	2
Escherichia coli ***	-	2 ***	2
*Pneumococci*	-	1	1
*Streptococcus sanguis*	-	1	1
*Staphylococcus dysgalactiae*	-	1	1
*Proteus mirabilis*	1	-	1
*Streptococcus oralis*	-	1	1
*Parvimonas micra*	-	1	1
*Actinobacter baumanii*	-	1	1
*Streptococcus acidominimus*	-	1	1
*Corynebacterium striatum*	-	1	1
*Staphylococcus hominis*	-	1	1
no organisms identified	8	1	9
mixed infections	2	6	8
multiresistent	-	3	3
atypical organisms	-	-	0

Identified organisms at implant removal. * one multiresistent Coagulase-negative Staphylococcus aureus (CNS). ** one “Methicillin-resistent Staphylococcus aureus” (MRSA). *** one “Extended-Spektrum Beta-Laktamases” organism (ESBL).

**Table 3:** Group B: Overview of all patients with positive cultures at the first sample collection.

Infecting Organism at Implant Removal	Change of Organism (y/*n*)	Organism at Sample Collection 1
*Coag. neg. S. (CNS)*	*n*	*Coag. neg. S. (CNS)*
*Coag. neg. S. (CNS)*	*n*	*Coag. neg. S. (CNS)*
*Coag. neg. S. (CNS)*	*n*	*Coag. neg. S. (CNS)*
*S. epidermidis*	*n*	*S. epidermidis*
*Coag. neg. S. (CNS) + Corynebacterium striatum*	*n*	*Coag. neg. S. (CNS)*
*Coag. neg. S. (CNS)*	y	*S. epidermidis*
*Coag. neg. S. (CNS)*	y	*Streptococci viridans*
*Methicillin-resistant S. aureus (MRSA)*	y	*Coag. neg. S. (CNS)*
*S. aureus*	y	*S. epidermidis*
*Propionibacterium acnes*	y	*S. caprae*
*S. dysgalactiae*	y	*S. haemolyticus*
*S. capitis*	y	*S. epidermidis*

All patients with a positive culture at first sample collection.

**Table 4:** Overview of all patients with positive culture at the second sample collection.

Infecting Organism at Explantation	Conversion of Organism y/n	Organism at First Sample Collection	Change of Organism y/*n*	Organism at Second Sample Collection
*Coag. neg. S. (CNS)*	-	negative	*n*	*Coag. neg. S. (CNS)*
*Streptococcus oralis*	-	negative	y	*S. epidermidis*
*Coag. neg. S. (CNS)*	*n*	*Coag. neg. S. (CNS)*	*n*	*Coag. neg. S. (CNS)*
*Coag. neg. S. (CNS)*	*n*	*Coag. neg. S. (CNS)*	*n*	*Coag. neg. S. (CNS)*
*Methicillin-resistant S. aureus (MRSA)*	y	*Coag. neg. S. (CNS)*	*n*	*Coag. neg. S. (CNS)*
*Coag. neg. S. (CNS) + Corynebact. striatum*	*n*	*Coag. neg. S. (CNS)*	*n*	*Coag. neg. S. (CNS)*
*Coag. neg. S. (CNS)*	y	*S. epidermidis*	y	*Enterobacter cloacae*
*S. dysgalac* *tiae*	y	*S. haemolyticus*	y	*S. epidermidis*

Infecting organisms detected during the second sample collection.

**Table 5:** Overview of infecting organisms found at re-implantation.

Infecting Organisms	Group A1	Group A2	Group B	Total
negative cultures	15	13	22	50
*Coag. neg. S. (CNS)*	6	2	3	11
*S. epidermidis*	-	-	3	3
*S. aureus*	-	1	1	2
*Propionibacterium acnes*	-	1	-	1
*Enterobacter aerogenes*	-	-	1	1

Frequency of detected infecting organisms during reintroduction.

**Table 6:** Infecting organisms at re-infection.

Patient	Infecting Organisms at						
	Implant Removal		Joint asp.		Re-Implantation	Re-Infection	
A1R1	*S. epidermidis + S. lugdunensis*		negative		*Coag. neg. S. (CNS)*	*S. aureus +* *ß-haemolytic Streptococci group B*	
A1R2	*Coag. neg. S. (CNS)*		negative		negative	*Coag. neg. SS (CNS)*	
A1R3	*Enterococci*		negative		negative	*S. aureus*	
**Patient**	**Infecting Organisms at**						
	**Implant Removal**		**Re-Implantation**		**Sampel Collection (SC)**	**Re-Infection**	
A2R1	*S. aureus*		negative		-	*S. aureus*	
A2R2	*Coag. neg. S. (CNS)*		*Coag. neg. S. (CNS) + S. aureus*		negative	*Coag. neg. S. (CNS)*	
**Patient**	**Infecting Organisms at**						
	**Explantation**	**SC1**	**SC2**	**SC3**	**Joint asp.**	**Re-Implantation**	**Re-Infection**
BR1	*Coag. neg. S. (CNS)*	*Coag. neg. S. (CNS)*	*Coag. neg. S. (CNS)*	negative	-	*Coag. neg. S. (CNS)*	*S. haemolyticus*
BR2	*Streptococcus viridans + Streptococcus* *acidominimus*	negative	-	-	-	negative	*S. aureus +* *S. epidermidis*
BR3	*Coag. neg. S. (CNS) + Corynebacterium striatum*	*Coag. neg. S. (CNS)*	*Coag. neg. S. (CNS)*	-	negative	negative	not known

**Table 7:** List of complications in all patients.

Complication	Group A	Group B	Total	Total (%)
re-infection	5	3	8	12.1%
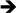 arthrodesis	1	1	2	3.0%
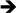 amputation	1	1	2	3.0%
wound healing problem	4	6	10	15.2%
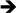 re-infection after wound healing problems	1	-	1	1.5%
arthrofibrosis	2	-	2	3.0%
acute artery occlusion	1	1	2	3.0%
deep vein thrombosis	1	-	1	1.5%
dislocation of the knee	1	-	1	1.5%
dislocation of the spacer	-	1	1	1.5%
transient ischämic attack	1	-	1	1.5%
temporarily cardiovacular problem	1	-	1	1.5%
fall with rupture of patellar ligament	1	-	1	1.5%
erysipelas	1	-	1	1.5%
decubitus	-	1	1	1.5%
haemolytic-uraemic symdrom	-	1		1.5%

Documented complications in all observed patients.

## Data Availability

The data presented in this study are available on request from the corresponding author due to privacy.
